# Quality of Life as reported by children and parents: a comparison between students and child psychiatric outpatients

**DOI:** 10.1186/1477-7525-8-136

**Published:** 2010-11-22

**Authors:** Thomas Jozefiak, Bo Larsson, Lars Wichstrøm, Jan Wallander, Fritz Mattejat

**Affiliations:** 1 The Norwegian University of Science and Technology (NTNU), Regional Centre of Child and Adolescent Mental Health, Department of Neuroscience, MTFS, N-7489 Trondheim, Norway; 2 Department of Child and Adolescent Psychiatry, St. Olavs Hospital Trondheim University Hospital, N-7433 Trondheim, Norway; 3 The Norwegian University of Science and Technology (NTNU), Department of Psychology, N-7491 Trondheim, Norway; 4 School of Social Sciences, Humanities, and the Arts, University of California, Merced, USA; 5 Department of Child and Adolescent Psychiatry, Universitätsklinikum Giessen und Marburg, Hans-Sachs-Str.6 D-35039 Marburg, Germany

## Abstract

**Background:**

During the recent decade, a number of studies have begun to address Quality of Life (QoL) in children and adolescents with mental health problems in general population and clinical samples. Only about half of the studies utilized both self and parent proxy report of child QoL. Generally children with mental health problems have reported lower QoL compared to healthy children. The question whether QoL assessment by both self and parent proxy report can identify psychiatric health services needs not detected by an established instrument for assessing mental health problems, i.e. the Child Behavior Checklist (CBCL), has never been examined and was the purpose of the present study.

**Methods:**

No study exists that compares child QoL as rated by both child and parent, in a sample of referred child psychiatric outpatients with a representative sample of students attending public school in the same catchment area while controlling for mental health problems in the child. In the current study patients and students, aged 8-15.5 years, were matched with respect to age, gender and levels of the CBCL Total Problems scores. QoL was assessed by the self- and parent proxy-reports on the Inventory of Life Quality in Children and Adolescents (ILC). QoL scores were analyzed by non-parametric tests, using Wilcoxon paired rank comparisons.

**Results:**

Both outpatients and their parents reported significantly lower child QoL on the ILC than did students and their parents, when children were matched on sex and age. Given equal levels of emotional and behavioural problems, as reported by the parents on the CBCL, in the two contrasting samples, the outpatients and their parents still reported lower QoL levels than did the students and their parents.

**Conclusions:**

Child QoL reported both by child and parent was reduced in outpatients compared to students with equal levels of mental health problems as reported by their parents on the CBCL. This suggests that it should be helpful to add assessment of QoL to achieve a fuller picture of children presenting to mental health services.

## Background

During the recent decade, a number of studies have begun to address Quality of Life (QoL) in children and adolescents (hereafter denoted as "children") with mental health problems in both general population [1,2] and clinical [3-19] samples. Whereas QoL has been found to vary with respect to child age and gender [14,20,21], generally children with mental health problems have reported lower QoL compared both to healthy children as well as those with a physical disorder [1,9]. Both children in child psychiatric outpatient [3,14] and inpatient [14] samples have shown significantly lower QoL compared to general population norms [22]. One study compared children with one of 10 chronic conditions, including different chronic physical diseases as well as psychiatric problems, to children in the general population [18]. Children with mental health problems reported lower QoL than those in the general population as well as those in several pediatric physical diseases. Children with attention deficit disorder have also been found to have a lower QoL compared to those with asthma [8], cancer and cerebral palsy [2], as well as healthy children [2,8,11]. Posttraumatic stress disorder, depression and alcohol use disorder have also been reported adversely to influence child QoL when compared to healthy controls [7].

However, only about half of the cited studies utilized both self and parent proxy report of child QoL. Moreover, while clinical studies have compared QoL in children with mental health problems and those in the general population, we found only one [8] that matched samples by age and gender while including children from the same geographic area. Still, this study used only parent proxy report. There are several limitations in using only child self report of QoL [23-25]*or *parent proxy report of child QoL [20,26-28]. Given different strengths and limitations with both QoL assessment approaches, both child self and parent proxy reports should be included when comparing clinical and healthy populations. The importance of using *both *child and parent proxy reports in child QoL research has also been emphasized by several authors [12,29,30].

Acknowledging the existence of different definitions of the multidimensional QoL concept pertaining to children [31], here we defined QoL in a narrow sense as "inner QoL". This refers to a subjective perceived well-being and satisfaction that can best be evaluated by the child, according to his/her own experience with regard to several life domains [32]. This concept is partly comprised of positive and negative affect as an emotional appraisal of health and life circumstances, as well as an emotional state that is determined by inter-personal aspects, temperament, etc. Although QoL is influenced by one's psychological state then, it can be distinguished from the concept of "psychopathology", which refers specifically to mental health problems/symptoms [31]. The American Psychiatric Association defines "psychopathology" as a manifestation of behavioural, psychological, or biological dysfunction in the individual [33]. It has also been shown that it is possible to improve QoL without improving psychopathology in referred child psychiatric outpatients, thereby providing evidence for the empirical difference between the concepts of "psychopathology" and "QoL" [6].

Assessment of psychopathology can be achieved in various ways, but the Child Behavior Checklist (CBCL) is the most commonly used paper-and-pencil method for this purpose. Part of Achenbach's Integrated System of Multi-informant Assessment [34], the parent-report CBCL can be complemented by the Youth Self-Report form (YSR), which has items largely corresponding to the CBCL. There is only moderate agreement between reports on the YSR and CBCL; agreement being highest on more observable Externalizing Problems (i.e. conduct problems and rule-breaking behaviour), and lowest on Internalizing Problems (i.e. depressive and anxiety dimensions) [34]. This underscores the importance of using both child and parent reports also with regard to the assessment of "psychopathology" whenever possible. However, the YSR, is only constructed for children aged 11 years and older, and thus would not be suitable for use in the current study focused on children from age 8.

Epidemiological and clinical research in child mental health has attempted to identify factors associated with referral to child mental health services. It is clear that factors beyond the child's symptom level play a role. Such factors are among others the impact of child symptoms on parents [35], family functioning [36], subjective expectations about psychiatric treatments, former experiences with services, attitudes and barriers towards such services [37], and external recommendation to seek services. However, it would be difficult to control for all these factors if one intends to examine whether QoL contributes to referral to psychiatric treatment. An alternative approach would be to investigate whether QoL explains psychiatric services needs beyond what is detected by a well-established, widely used, reliable, valid and comprehensive assessment instrument for mental health problems/psychopathology in children. To our knowledge this question has never been examined in relation to referral to child psychiatric services. If QoL measurement in children and adolescents referred to mental health services adds important information to their referral status that is not detected by the assessment of mental health problems this would suggest that it will be useful to assess QoL to achieve a fuller picture of children presenting to mental health services.

In any comparison study between clinical and healthy populations there is the question of "how healthy" should the healthy reference population be? The general population may include children who are receiving services in alternative settings or have a history of mental health referral. Should these children in the general population be excluded or not? Because either approach has advantages and disadvantages, we present analyses both with and without exclusion of such children.

The overall aims of the present study thus were to investigate the question whether QoL assessment could identify psychiatric health services needs over and beyond that provided by the CBCL. Self- and parent proxy reports of QoL in a sample of referred child psychiatric outpatients, aged 8-15.5 years, were compared with a sample of children, matched for age, gender and levels of total emotional/behavioural problems, attending schools in the same health care catchment area. The following specific research questions were addressed in this explorative study:

(1) Will child psychiatric outpatients and their parents report significantly lower levels of QoL compared to students and their parents?

(2) When matched for levels of emotional and behavioural problems reported by parents on the CBCL, will the difference in QoL between outpatients and students remain?

(3) When excluding students who had received help due to mental problems or learning difficulties (and their matched outpatients) will the difference in QoL between outpatients and students remain?

## Methods

### Sample selection and subjects

#### Clinical sample

From July 2003 until December 2005, children and adolescents aged 8-15.5 years, who had been referred consecutively for the first time and had at least two visits at three outpatient clinics of the Department of Child and Adolescent Psychiatry/St. Olav University Hospital in the Norwegian county of Sør-Trøndelag, were invited to participate in the study. The criterion of a minimum of two visits was chosen to exclude children who had been referred because of other reasons than treatment (e.g. mental health evaluation in a somatic ward or from social service). Children and parents with insufficient competence in the Norwegian language (11 refugees) were also excluded.

Of 501 eligible outpatients fulfilling these inclusion criteria, parents did not give informed consent for 82 (16.4%), and for another 74 (14.8%), the clinical staff did not follow the procedures of the research protocol. Thus, 345 children (141 girls and 204 boys, mean age 11.6 years; SD = 2.2) were included in the final study sample, with an overall response rate of 68.9% (out of 501 eligible outpatients). For 327 (94.8%) of the 345 patients, at least one parent (306 mothers and 21 fathers) completed the parent proxy form of the Inventory of Life Quality in Children and Adolescents (ILC) (see below), and 293 of the patients completed the child self-report form.

The final outpatient sample showed a slightly skewed urban-rural ratio (1:1.2) in favour of rural areas as compared to the 156 patients who dropped out (1.2:1) (χ^2 ^(1) = 5.06, p < 0.05). However, there was no significant difference between included patients and dropouts in terms of child living condition (with one or two biological parents) or psychosocial functioning as measured by the ICD-10 axis 6 scale (see below). Type of problems described in the physician referral for both *included patients *and *dropouts *are shown in Table 1, but there were no significant differences.

**Table 1:** Type of problems in the physician referrals for included outpatients and dropouts by four subgroups

Group of problems	Physician's reason of referral	Included patients (%) **n = 331**^**a**^	Drop-outs (%) **n = 148**^**b**^
Emotional problems	Depressive, suicidal, anxious, compulsive, eating disorder	42.0	48.6
Behavioural problems	Hyperactivity/attention and conduct problems	44.4	35.8
School problems	Learning, language- and speech problems and school-phobia	4.8	6.8
Other	Autistic or psychotic symptoms, visual/auditory problems	8.8	8.8

None of the observed differences were significant by Pearson Chi-Square.

^a^345 totally included outpatients and ^b^156 drop-outs; the difference to n is due to "no problem specified" on physician referral sheet.

#### School sample

From September 2004 until November 2005, 1997 students (990 girls, 1007 boys) aged 8-16 years were included by random cluster sampling in a representative school sample from the same county, with a response rate of 71.2% for eligible students. Two previous studies on QoL in school-children have been published based on this school sample, thereby establishing psychometric properties of the Norwegian version of the used QoL instrument, comparing child and parent proxy reports and investigating changes of QoL in school-children during a six-month period [20,26]. Further, one study of children with obesity referred to a pediatric clinic was published also including a portion of these data [38]. For the present study one geographic region of the county had to be excluded because it was not included in the health care catchment area covered by the participating outpatient clinics. Thus, a final school sample of 1729 students was used in the analyses. Parent report was available for 1513 students, of whom 119 (8%) answered positively to the following question: "Has your child received any help during the last year due to mental health problems or learning difficulties?"

#### Assessment procedures

A research assistant registered all outpatients who were referred for the first time to the outpatient clinics and fulfilled the inclusion criteria. The therapists who met with the patient and his/her parent informed about the project and handed out information letters. They stressed informant confidentiality and responded to questions. The therapists also decided if the patient fulfilled any exclusion criteria and scored the patient on the psycho-social functioning scale (see below). Participating outpatients aged 12 years or older and their parent(s) completed the questionnaires independently. For children aged 11 years or younger, a structured administration of the ILC (see below) was conducted by the therapists where items are read aloud and response choices recorded.

For the school-sample, one teacher at each school was appointed as a project coordinator and given information about the research project and procedures for collecting the data. The coordinator informed the students about the project and also sent a standard information letter to their parents. The principal investigator or a research assistant administered the questionnaires to the students. They stressed informant confidentiality, responded to questions, and read questions aloud for students with reading problems and all pupils in the 4^th ^grade [20].

### Measures

#### Socio-demographic and clinical information

In the school sample information on child age and sex were given by both child and parent in the questionnaires. Information about number of caregivers was given by parents. For the patients such information was obtained through the electronic medical record system.

#### The Inventory of Life Quality in Children and Adolescents (ILC)

A Norwegian translation [20] of the ILC [39] for children, adolescents, and their parents was used to assess QoL over the past week. It includes one item for global evaluation of QoL and six items addressing subjectively reported well-being in regard to the child's physical and mental health, perception of activities when the child is alone, perceived relationship to friends and family as well as to school. Each item is rated on a 1 - 5 scale (1 = Very good, 5 = Very bad). Two scores were used herein following standard procedures for scoring the ILC [39]: (1) The ILC 0-100 score, representing a total QoL score, is obtained by summing the rating values across the seven items and transforming them into a 0-100 scale (0 = Very low QoL, 100 = Very high QoL). (2) A Problem score 0-7 is obtained by summing each of the seven items after they had been dichotomized, such that ratings of 1 or 2 represented "no problem", and ratings of 3, 4 or 5 represented "problem present". This ILC Problem 0-7 scale score indicates the number of life domains perceived as problematic (0 = no problem on any life domain, 7 = problems on all life domains).

We used the ILC because in this study it has certain advantages compared to other QoL measures available for children and adolescents. It is a generic measure which has been developed for use in child psychiatry, and includes the item "perception of activities when the child is alone". It is short and therefore easy to use in a clinical setting. Further, it also provides a simple sum score (Problem score 0-7) indicating the amount of QoL domains perceived by the child as problematic. Finally, the ILC has shown a moderate convergent validity with the KINDL (Questionnaire for Measuring health-related Quality of Life in children and adolescents), a well established measure, both in the German and Norwegian version (r = .65 and r = .69, respectively) [20,22,40]. However, the KINDL has not the advantages described above in regard to the present study. The Norwegian version of the ILC has shown satisfactory internal consistency (alpha for child-report from 0.64 to 0.81 for the 4^th ^to 10^th ^grade, respectively; alpha for parent proxy version = .80), comparable to the ILC original version and the KINDL, as well as two-week test-retest reliability of 0.86 (ICC) for the ILC 0-100 score [20].

#### The Child Behavior Checklist (CBCL)

The CBCL [34] is part of an integrated system of multi-informant assessment of children and adolescents that has been used extensively in research and clinical practice. Parents are asked to report on children's emotional and behavioural problems over the preceding 6 months. Each item is rated on a 0-2 scale (0 = Not True, 1 = Somewhat or Sometimes True, or 2 = Very True or Often True). Here, the Total Problems scale consisting of 118 items of the school-age form for 6-18 year-old children was used (total score range of 0-236). Further, Internalizing and Externalizing Problems scale scores were also calculated. The Norwegian translation of the CBCL has shown satisfactory predictive, discriminant and convergent validity [41].

#### The ICD-10 Global assessment of psycho-social functioning level

The ICD-10 [42] axis 6 is used by the therapist to assess the psycho-social functioning of the child as part of clinical routine assessment. It consists of eight response choices, where 0 = Excellent psychosocial functioning and 8 = Extreme and pervasive social dysfunctioning.

#### Ethics

Before patient participation in the study, parents had to give their written consent. The Norwegian Ethical Committee of Medical Research and the Norwegian Data Inspectorate approved of the study.

### Statistical analysis

#### Matching of outpatient-student pairs

Children who had complete data on the ILC self-report in both clinical and school samples were matched by sex and age group (8-9.5; 10-11.5, 12-13.5 and 14-15.5 years), resulting in a matched sample of 292 patient-student pairs (N = 584) (see Table 2). Parents' QoL proxy reports were available for 291 of these pairs. Parents of outpatients reported significantly (t(281) = 18.89, p < 0.001) more emotional and behavioural Total Problems on the CBCL than did those of students (M = 49.1, SD = 25.9 vs. M = 15.9, SD = 16.3 respectively).

**Table 2:** Outpatients and students matched by age, sex and levels of emotional and behavioural problems

Age-group	8 - 9.5 years	10 - 11.5 years	12 - 13.5 years	14 - 15.5 years	Total pairs
*Matched on sex and age*					
**Girls**	20	32	31	34	117
**Boys**	49	53	35	38	176
**Total**	69	85	66	72	292
*Matched on sex, age and level of emotional and behaviour problems^1^*					
**Girls**	4	11	9	8	32
**Boys**	18	24	17	13	72
**Total**	22	35	26	21	104

^1^Total Problems score on the CBCL

Next, CBCL Total Problems scores were calculated for both samples. In the school sample, CBCL data from 1503 parents were available. Total Problems scores above the 90^th ^percentile (score > 31), were divided into five-point intervals. Outpatients with Total Problems scores within the same intervals were individually randomly matched with students of the same sex and age subgroup, resulting in 104 patient-student pairs (N = 208) (see Table 2). Parent proxy reports on child QoL were also available for 102 of these pairs. After matching, no significant difference between outpatient and student samples was found on the Total (M = 48.0, SD = 13.8 and M = 47.7, SD = 14.0), Externalizing (M = 14.4, SD = 7.2 and M = 14.5, SD = 7.4) and Internalizing (M = 13.4, SD = 8.0 and M = 13.5, SD = 6.5; respectively) Problems Scores. Likewise, there was no significant difference on the Total Problems scores between outpatients in the age-, sex- and Total Problems score-matched-sample and those remaining in the outpatient sample who had not been matched with students.

#### Analysis Procedures

Missing values on standardized measures were substituted by using Expectation Maximization (EM) procedures. In the clinical sample missing items were 0.3-1.7% on the ILC child self-report, 0.0-0.9% on the ILC parent proxy report and 0.0-4.6% on the CBCL. In the school sample the corresponding values were 0.3-1.1%, 0.3-0.6% and 0.0-1.9%; respectively. Chi-square statistics were used in comparing proportions between groups. Differences in group means were compared by dependent and independent t-tests for continuous variables. Though, because of significant skewness and kurtosis for most of the ILC scores analyzed herein, non-parametric tests, using Wilcoxon paired rank comparisons were reported. All analyses were also completed with parametric tests of the means by dependent t-tests with identical findings to those reported for the non-parametric tests, except for the difference between patient and student on the ILC 0-100 scores for the subgroup "Parent of student did not report child had received help in past 12 months". For this difference a p value of 0.06 was obtained by the parametric test. Correlations were calculated by Pearson r and Spearman's rho. An alpha level of p < 0.05 indicated statistical significance.

## Results

### Preliminary analysis

The Spearman's correlations between the parent QoL proxy report (ILC 0-100 scores) and the CBCL Total Problems scores were rho = -.66 (p < 0.01; n = 312) in the clinical sample and rho = -.61 (p < 0.01; n = 298) in the school sample. The correlation between patient QoL self-report and the CBCL Total Problems scores were rho = -.23 (p < 0.01; n = 276) in the clinical and rho = .-25 (p < 0.01; n = 297) in the school sample.

### Comparisons between age and gender matched samples

Based on self-report, patients scored significantly (z = -8.170, p < 0.001, Wilcoxon) lower than did students on the ILC 0-100 scale (for details, see Table 3) and also reported significantly (z = -8.319, p < 0.001, Wilcoxon) more problematic QoL domains than did the students on the ILC Problem 0-7 scale. Similarly, parents of outpatients evaluated their children's QoL levels significantly (z = -12.042, p < 0.001, Wilcoxon) lower than did those of students on the ILC 0-100 scale (for details, see Table 3). Parents of outpatients also reported significantly (z = -11.424, p < 0.001, Wilcoxon) more problematic QoL domains than did those of students on the ILC Problem 0-7 scale (see Table 3).

**Table 3:** Median differences in QoL by child- and parent proxy reports in age-and sex-matched outpatient-student pairs.

	Patient Median	Student Median	N (pairs)	Patient's parent Median	Student's parent Median	N (pairs)
**ILC 0-100**	71.4 ***	**82.1**	292	67.9 ***	**85.7**	291
**ILC Problem-score 0-7**	2.0 ***	**1.0**	292	3.0 ***	**0.0**	291

***p < 0.001 (Wilcoxon Signed Ranks test)

### Controlling for level of emotional and behavioural problems

Based on analysis of data from 104 matched pairs, outpatients reported significantly (z = -2.409, p < 0.05, Wilcoxon) lower QoL on the ILC 0-100 scale than did the students (see Table 4). They also reported significantly (z = -2.731, p < 0.01, Wilcoxon) more problematic QoL domains than did the students on the ILC Problem 0-7 scale (see Table 4). The comparison of the parent reports for the matched sample of students and outpatients showed no significant differences in QoL either on the ILC 0-100 or ILC Problem 0-7 scale (Table 4).

**Table 4:** Median differences in QoL by child and parent proxy report in outpatient-student pairs^1, 2^

	Patient Median	Student Median	N (pairs)	Patient's parent Median	Student's parent Median	N (pairs)
*Total sample*						
**ILC 0-100**	71.4*	**78.6**	86	67.9	67.9	102
**ILC Problem-score 0-7**	2.0**	**1.0**	86	3.0	3.0	102
*Parent of student did not report child had received help in past 12 months*						
**ILC 0-100**	71.4*	**78.6**	54	67.9**	**75.0**	66
**ILC Problem-score 0-7**	2.0*	**1.0**	54	3.0**	**2.0**	66
						

^1 ^in the outpatient and school samples, matched for sex, age and levels of emotional and behavioural problems.

^2 ^shown both for the total sample and when excluding student-patient pairs where student has received help recently.

* *p *< 0.05, ** *p *< 0.01 (Wilcoxon Signed Ranks test).

### Excluding student-patient pairs where student has received help recently

Thirty-three of the students were reported by a parent to have received professional help in past 12 months due to learning difficulties or mental health problems. When those 33 student-patient pairs were excluded from the analysis, the differences between patient and student reports on the ILC 0-100 and ILC Problem 0-7 scale remained significant (z = -2.003, p < 0.05 and z = -2.161, p < 0.05, respectively, Wilcoxon, see Table 4). Likewise, parents of outpatients reported significantly (z = -3.187, p < 0.01, Wilcoxon) lower child QoL on the ILC 0-100 scale (see Table 4) than parents of students. They also reported significantly (z = -3.152, p < 0.01, Wilcoxon) more problematic life domains than did the students on the ILC Problem 0-7 scale (see Table 4).

## Discussion

In the present study, comparing QoL between psychiatric outpatients and regular students recruited from the same catchment area and during comparable time intervals and matched for sex and age, we found outpatients to report, and be reported by their parents with, significantly lower QoL than did students. Moreover, when also matching for levels of emotional and behavioural problems in the two different groups, the outpatients still reported lower QoL levels than students. Finally, when also excluding students in the general population who had received services in alternative settings or had a history of mental health referral the outpatients still reported, and were reported with, significantly lower QoL levels than students.

### Child report of QoL among outpatients and students with equal levels of emotional and behavioural problems

Our results indicate that QoL self-report could identify psychiatric health services needs that are not detected by a well-established, widely used, reliable, valid and comprehensive assessment instrument for mental health problems/psychopathology in children. The results showed that even when the outpatients were matched with students for levels of emotional and behavioural problems, they still reported significantly lower QoL levels than did the students. Therefore, QoL could add important information about the child's perceived well-being beyond that provided by only considering mental health or psychopathological aspects, at least when measured as parent reported emotional and behavioural problems on the CBCL.

The child's QoL as measured by the ILC refers to subjective well-being and satisfaction, according to his/her experience in several life domains such as in the family, with peers, in school, in activities when the child is alone, and in regard to his/her physical and mental health, together with a global rating of wellbeing [32,39]. Our results suggest that given two children with equal levels of mental health problems, QoL measurement would add important information about the patient. The reduced QoL experience in outpatients, compared to non-patients with equal mental health problems, would suggest referred children have more psychiatric health services needs that cannot be explained solely by their mental health problems.

In addition to specific information about the child's QoL as mentioned above, the ILC also provides information whether certain life domains are perceived as problematic or non-problematic. Our results show also that even when the outpatients were matched with students for levels of emotional and behavioural problems they reported a larger number of problematic life domains than did the students. Even if the child perceives a markedly reduced QoL in one or two life domains, areas where there are no problems can represent potential strengths for the child. The clinician may find it useful to build on such assets in treatment planning. Thus, assessment of QoL using an instrument such as the ILC can provide a fuller picture of children's needs and opportunities when presenting to mental health clinics.

### Parent report of QoL among outpatients and students with equal levels of emotional and behavioural problems

Parents of outpatients reported lower child QoL levels when compared to parents of students in the general population, even when controlling for levels of emotional and behavioural problems in the child (as reported by the parents). However, this significant difference was only observed after exclusion of those patient-student pairs where the student's parent had answered positively to the question: "Has your child received any help during the last year due to mental health problems or learning difficulties?"

Interpretation of this finding is not straightforward. These parents of children in the school population obtaining help because of child mental problems or learning difficulties might be in a quite different situation than those of children in the school population, who have not obtained any help (most of them probably because there is no need for any help).

Parents of students could have received help for their child from community primary care services but they actually may have perceived this help as inadequate due to limited special mental health service capacities. They therefore could have rated child QoL as lower than did parents of healthy children, thereby reducing the difference between patient's and student's parent proxy reports. Without excluding the "having received help" group from the analysis parents of outpatients and students did not report any significant difference on child QoL (see Table 4). We feel this finding further reinforces the need for both child and parent proxy report in QoL assessment because it seems that obtaining help for one's child because of mental problems or learning difficulties affected the student's parent proxy, but not the child reports in the present study.

Not excluding students who had received help lead to non-significant difference between parent report of students and patients and might be interpreted mistakenly. Given non-significant differences between parent proxy reports and significant differences between self-reports, the additional discrimination of QoL measures in addition to the parent reported CBCL could simply be interpreted as a result of the self-report perspective. However, we found that parent-report QoL measures *as well as *self-report could add important information about the child's perceived well-being beyond that provided by only considering measures of mental health or psychopathology, at least when considering parents in the school population who's child did not received help recently.

### Help provided in the school-sample and the problem of double participation

Identification of children in school-samples is typically impossible due to ethical reasons for protecting anonymity, as it was in the present study. Some students might therefore have participated both in the school-based and clinical sample when targeting the same catchment area, which could bias the results. We do not know exactly where these students had received help, such as from either primary care services for students (i.e. general practitioners, nurses, school psychologists) or our specialized child psychiatric service. About 2-3% of the county's total child population, aged 0 to 18 years, received specialized psychiatric services (Annual Reports for 2003; BUP-Aarsmelding St. Olav Hospital in Trondheim). For children 8-15.5 years this figure is estimated somewhat higher or about 4%. Eight percent of the students (aged 8-15.5 years) in the total school-sample were reported by their parents to have received help for their child. Thus, up to one-half of these students who received some kind of help might have had contact with our specialized psychiatric service. When we matched patients and students on the CBCL, 33% of the student's parents reported having received help. Therefore, roughly, at maximum one half of them (15%, N = 15) could have participated in both the school and clinical sample. However, as discussed above, we conducted analyses both with and without students who had received such help, thereby partly controlling for confounding by overlapping samples.

### Strengths and Limitations

When controlling for sex and age we found both outpatients and their parents to report lower QoL than did students and their parents. These results are consistent with outcomes of other clinical studies examining QoL by child [3,7,9,14,18] and parent proxy [3,8,9,11,18] report, thereby validating our study. Other strengths of the present study were that we compared outpatients with students matched for sex and age, and that both child and parent proxy reports of QoL were obtained. In addition all subjects resided in the same health care catchment areas and assessments were carried out in closely related time periods in both groups. When controlling for emotional and behavioural problems, the observed additional discriminatory power provided by the ILC might have been contained in the CBCL, but might not be accessible due to matching by using only the Total Problem score. However, after the matching procedure we found no significant differences neither on the Externalizing nor the Internalizing subscales, thereby partly controlling for any such confounding.

However, there were several limitations with the study. The ILC being brief, does not address psychosocial functioning in detail. Although, the concept of "inner" QoL indirectly also reflects the child's *subjective perception *of his/her functioning on different life domains, "psychosocial functioning" often refers to more "objective" aspects of the individual's life. "Psychosocial functioning" is important for both the QoL and mental health perspective, but can best be evaluated from an external perspective [32], for example by achievements in school, size and number of contacts with social network, and/or diagnostic ratings of psychosocial functioning by a clinician. However, the resources available for the present study did not permit such assessment, especially not in the student comparison group.

Another limitation is that we did not include self-reports of mental health, such as with the YSR, even though we included the child perspective in QoL measurements. We did not use the YSR (which is constructed for children aged 11 years and older) in the present study because that would considerably reduce the size of our present clinical sample leading to reduced power corrupting the results. Further, there is no other comprehensive, reliable, valid and well-established instrument for assessing mental health by self-report for the youngest children. However, research on mental health and QoL in the younger age group is important because school services play a central role in providing support and early detection of children who need to be referred to mental health services [43].

Furthermore, the information on child emotional and behavioural problems was obtained by questionnaires, and not by semi-structured clinical interviews conducted by clinical professionals, which are acknowledged as the gold standard. However, interviews are difficult to incorporate in epidemiological studies.

Lastly the present study was limited to one county in central Norway. Although this included children from rural, semirural as well as urban areas, the population is highly homogenous in race/ethnic make up. Moreover, socioeconomic status is more restricted in Norway than in most countries.

## Conclusion

In this explorative study, child QoL reported both by child and parent was reduced in outpatients compared to students with equal levels of mental health problems as measured by parent-report on the CBCL. This suggests that it could be helpful to add assessment of QoL to achieve a fuller picture of children presenting to mental health services.

## Abbreviations

CBCL: The Child Behavior Check List; ILC: The Inventory of Life Quality in Children and Adolescents; ICD-10: The International Classification of Mental and Behavioural Disorders; ILC 0-100: ILC Life Quality score (range 0-100); ILC Problem score 0-7: indicating the number of life domains perceived as problematic (range 0-7); QoL: Quality of Life; YSR: The Youth Self Report.

## Competing interests

The authors declare that they have no competing interests.

## Authors' contributions

TJ contributed to the study design, data collection, statistical analysis, interpretation of the data and the drafting of the paper. BL contributed to the study design, statistical analysis, interpretation of the data and the revising of the manuscript. LW made contribution to the study design, statistical analysis, interpretation of the data and the revising of the manuscript. JW contributed to statistical analysis, interpretation of the data and the revising of the manuscript. FM is one of the original authors of the ILC, and made a contribution to the translation process of the Norwegian ILC, statistical analysis and the revision of the manuscript. All authors have read and approved the final manuscript.
